# Changes in Life’s Essential 8 and risk of cardiovascular disease in Chinese people

**DOI:** 10.1093/eurpub/ckae063

**Published:** 2024-04-04

**Authors:** Wenjuan Li, Aijun Xing, Sander Lamballais, Wenqi Xu, Shuohua Chen, Shenghua Zhou, Shouling Wu, Zhangling Chen

**Affiliations:** The School of Clinical Medicine, North China University of Science and Technology, Tangshan, Hebei, China; Department of Cardiology, Kailuan General Hospital, Tangshan, Hebei, China; Department of Cardiology, Kailuan General Hospital, Tangshan, Hebei, China; Department of Clinical Genetics, Erasmus MC, University Medical Center Rotterdam, Rotterdam, The Netherlands; The School of Clinical Medicine, North China University of Science and Technology, Tangshan, Hebei, China; Department of Cardiology, Kailuan General Hospital, Tangshan, Hebei, China; Department of Cardiology, Kailuan General Hospital, Tangshan, Hebei, China; Department of Cardiovascular Medicine, The Second Xiangya Hospital, Central South University, Changsha, Hunan, China; Department of Cardiology, Kailuan General Hospital, Tangshan, Hebei, China; Department of Cardiovascular Medicine, The Second Xiangya Hospital, Central South University, Changsha, Hunan, China; Hunan Key Laboratory of Cardiometabolic Medicine, Changsha, Hunan, China; FuRong Laboratory, Changsha, Hunan, China; Department of Epidemiology, Erasmus MC, University Medical Center Rotterdam, Rotterdam, The Netherlands

## Abstract

**Background:**

The American Heart Association recently released an updated algorithm for evaluating cardiovascular health—Life’s Essential 8 (LE8). However, the associations between changes in LE8 score over time and risk of cardiovascular disease (CVD) remain unclear.

**Methods:**

We investigated associations between 6-year changes (2006–12) in LE8 score and risk of subsequent CVD events (2012–20) among 53 363 Chinese men and women from the Kailuan Study, who were free from CVD in 2012. The LE8 score was calculated based on eight components: diet quality, physical activity, smoking status, sleep health, body mass index, blood lipids, blood glucose and blood pressure. Multivariable-adjusted Cox proportional-hazards models were used to estimate hazard ratios (HRs) and 95% confidence intervals (CIs).

**Results:**

We documented 4281 incident CVD cases during a median of 7.7 years of follow-up. Compared with participants whose LE8 scores remained stable in a 6-year period, those with the large increases of LE8 score over the 6-year period had a lower risk of CVD, heart disease and stroke in the subsequent 8 years [HRs and 95% CIs: 0.67 (0.64, 0.70) for CVD, 0.65 (0.61, 0.69) for heart disease, 0.71 (0.67, 0.76) for stroke, all *P*_trend_ < 0.001]. Conversely, those with the large decreases of LE8 score had 47%, 51% and 41% higher risk for CVD, heart disease and stroke, respectively. These associations were consistent across the subgroups stratified by risk factors.

**Conclusions:**

Improving LE8 score in a short- and moderate-term was associated with a lower CVD risk, whereas decreased LE8 score over time was associated with a higher risk.

## Introduction

Cardiovascular disease (CVD) remains the leading cause of death worldwide, accounting for 32% of all global deaths.^1^ It is, therefore, essential to develop population-wide preventive approaches to reduce the risk and burden of CVD. Recently, the American Heart Association (AHA) released an updated concept for evaluating cardiovascular health (CVH), called Life’s Essential 8 (LE8), aiming to improve CVH in general populations.^2^

Compared to the earlier developed Life’s Simple 7 (LS7),^3^ the LE8 is updated and enhanced by including an additional new component—sleep. The LE8 thus includes the eight most important predictors of CVH: diet quality, physical activity, body mass index (BMI), smoking status, sleep health, blood pressure, blood glucose and blood lipids.^2^ Furthermore, the scoring system of the LE8 has been expanded, with each LE8 component varying from 0 to 100 points to detect interindividual differences in populations and changes in CVH over time,^2^ while the LS7 system assigns a score from 0 to 2 for each component (0 = poor, 1 = intermediate, 2 = ideal).^3^

Several recent epidemiological studies have observed consistent inverse associations between the LE8 score and subsequent risk of CVD events.^4–9^ However, most of these studies only considered the baseline measurement of adherence to the LE8 score. In real life, a person’s eating behavior, lifestyle and health conditions are likely to change over time, and a single measurement of the LE8 score would not adequately capture these dynamic changes over time. From a public health and clinical standpoint, it is critical to examine the associations of changes in adherence to the LE8 score over time with subsequent risk of CVD. This would lead to a more comprehensive understanding of the potential impact of adherence to the LE8 score on risk of CVD. Moreover, evaluating the associations of changes in adherence to the LE8 score over different time periods (e.g. 2-year, 4-year, 6-year and 8-year changes) with CVD risk will also help determine how quickly such changes in the LE8 score may impact CVD risk. Yet, to our knowledge, no studies have examined the associations between changes in adherence to the LE8 score over time and subsequent risk of CVD.

To fill these critical knowledge gaps, we prospectively investigated the associations between changes in LE8 score over time and subsequent risk of CVD, including heart disease and stroke in a large ongoing Chinese prospective cohort study: the Kailuan Study. Data on diet, lifestyle and health conditions were collected biennially in the Kailuan Study from 2006 through 2020. This enabled us to evaluate the associations between 6-year changes (2006–12) in adherence to the LE8 score and subsequent risk of CVD (2012–20). In secondary analyses, we also examined shorter-term (2-year, 4-year) changes and longer-term (8-year) changes in LE8 score in relation to CVD risk.

## Methods

The Kailuan Study is an ongoing prospective cohort study of participants from the Kailuan Group, a coal mining company in Tangshan, China. The study design and procedures have been previously described in detail.^10–12^ Briefly, 101 510 participants aged 18 years or older were included from 11 affiliated hospitals and clinics of the Kailuan Group in 2006. The study was subsequently extended by including new participants in 2008 (*n* = 24 540), 2010 (*n* = 9118), 2012 (*n* = 17 981), 2014 (*n* = 9088), 2016 (*n* = 4112) and 2018 (*n* = 4737). By the end of 2018, the Kailuan Study included a total of 171 086 participants aged 18 years or older. Upon entering the study, participants received a series of questionnaire assessments, clinical examinations and lab tests in the 11 affiliated hospitals and clinics every 2 years. All employees and retirees in the Kailuan Group were obliged to enroll in the Urban Employee Basic Medical Insurance, and drug treatment could be partly reimbursed by health insurance.

For this study, the initial cycle was set at 2006, the baseline was set at 2012 (changes in the LE8 score were calculated from 2006 through 2012), and the end of follow-up was 2020. We excluded participants who had a history of CVD, including heart disease [e.g. myocardial infarction (MI), atrial fibrillation (AF) and heart failure (HF)] and stroke at or before the baseline in 2012, or those missing information regarding the LE8 score. The final analysis included 53 363 participants (Supplementary figure S1). The Kailuan Study was approved by the Ethics Committee of the Kailuan Medical Group, and all participants provided written informed consent.

### Exposure assessment

According to the AHA’s LE8 algorithm, we created LE8 score using the following eight components: four lifestyle factors (diet health, physical activity, smoking and sleep health) and four health factors [BMI, non-high density lipoprotein cholesterol (non-HDL cholesterol), blood glucose and blood pressure] (Supplementary table S1).^2^ The information on diet, physical activity, smoking and sleep was collected using standardized self-reported questionnaires every 2 years since 2006. Dietary salt intake, tea consumption and fatty food consumption were used as a surrogate measure for diet quality, which have been previously reported to be related to CVD risk in Chinese people.^13^^,^^14^ Weight, height, blood lipids, blood glucose and blood pressure were measured at the medical examination center of the Kailuan Group every 2 years since 2006. We calculated BMI by dividing weight (kg) by the square of height (m). Blood samples were collected in the morning following an 8–12-h overnight fast at each visit. The fast blood glucose, total cholesterol, low-density lipoprotein cholesterol and HDL cholesterol were measured using a Hitachi 7600 autoanalyzer (Tokyo, Japan) at the central laboratory of Kailuan General Hospital. Supplementary table S1 presents the detailed information and scoring algorithm for each component. We averaged the component scores to derive the overall LE8 score, ranging from 0 (worst health) to 100 (best health).^3^ To explore whether the associations of changes in LE8 score and the outcomes were robust in different time periods, we calculated 6-year changes in LE8 score from 2006 to 2012 as the main exposures to evaluate their associations with subsequent risk of CVD. We assessed 2-year (2006–08), 4-year (2006–10) and 8-year (2006–14) changes in LE8 score and examined the associations in a secondary analysis.

### Ascertainment of CVD

Incident CVD was defined as heart disease (including MI, AF and HF) and stroke. The information of CVD diagnosis was obtained from the municipal social insurance institution and the hospital discharge register, which covered all the Kailuan Study participants, and the information was updated annually during the follow-up period. For all suspected CVD events, an expert panel reviewed the annual discharge records for confirmation of diagnosis. MI diagnosis was determined by a combination of the individual’s clinical symptoms, electrocardiography findings and dynamic changes in myocardial enzymes, in line with WHO’s Multinational Monitoring of Trends and Determinants in CVD criteria.^15^ AF diagnoses were retrieved from the same discharge registers and from resting electrocardiogram during each survey. The electrocardiogram and diagnosis were completed by two professional electrocardiologists, according to the European Society of Cardiology guidelines.^16^ Incident HF was identified based on clinical symptoms, echocardiography, chest X-ray and electrocardiography, as per the criteria of the European Society of Cardiology.^17^ Stroke was diagnosed following the WHO criteria, based on neurological signs, clinical symptoms and neuroimaging tests, including computed tomography or magnetic resonance imaging.^18^

### Covariates

Information on anthropometric, lifestyle and socioeconomic factors was obtained through biennial questionnaires administered every 2 years starting in 2006. The variables included age, sex, alcohol consumption, education level, income, marital status and family history of CVD.

### Statistical analyses

We categorized 6-year changes in LE8 score into quintiles from the large decrease (quintile 1) to the large increase (quintile 5). We used Cox proportional-hazards models to estimate the hazard ratios (HRs) and 95% confidence intervals (CIs) associated with changes in the LE8 score and CVD incidence. To account for potential confounding, we adjusted for initial age (continuous), sex (male, female) and initial LE8 score (continuous) in model 1. We further adjusted for education (illiteracy or elementary, middle school, college or university), initial income (< median, ≥ median) and changes in income (< median always, ≥ median always, change from < median to ≥ median, change from ≥ median to < median), initial marital status (yes, no) and changes in marital status (always single, always married, change from married to single, change from single to married), initial alcohol-drinker (yes, no) and changes in alcohol-drinker (always drinker, always non-drinker, change from drinker to non-drinker, from non-drinker to drinker) and family history of CVD (yes, no) in model 2. Tests for trend were computed by assigning the median value to each quintile. We estimated the risk of CVD per 10-point increase in the LE8 score by treating the scores as continuous variables. To test for potential non-linearity of the associations of changes in the LE8 score with CVD risk, we used restricted cubic spline regression analyses with four knots to flexibly model the associations. We also conducted a joint analysis of baseline LE8 score and 6 years later LE8 score with CVD risk. In secondary analyses, we examined associations of 2-year (2006–08), 4-year (2006–10) and 8-year (2006–14) changes in LE8 score with subsequent risk of CVD.

Furthermore, we stratified analyses by age, sex, follow-up time, alcohol consumption, initial LE8 score and family history of CVD. We used a Bonferroni-corrected *P* values threshold (0.05/6 = 0.008) to account for multiple comparisons in the interaction tests of the stratified analyses.

### Sensitivity analyses

We conducted several sensitivity analyses to test the robustness of our findings. First, we conducted 2-year lag analyses to minimize reverse causation. Second, we repeated the analyses by excluding CVD events diagnosed in the first 2 years of the follow-up. Third, we additionally adjusted for antihypertensive medication use, lipid-lowering medication use and glucose-lowering medication use. Fourth, we further excluded participants with cancers at baseline and follow-up. Fifth, we created 6-year changes in LS7 score, analyzed its associations with CVD risk, and compared the effect sizes of the associations of changes in LE8 score and LS7 score. Sixth, we examined the associations of changes in each component score of LE8 score with risk of the outcomes. Finally, the capacity of the model of changes in LE8 score with adjusted covariates for predicting CVD risk was examined via area under the receiver-operating characteristics curves analysis.

Missing values on covariates were accounted for using multiple imputations of chained equations.^19^ As the percentages of missing values ranged from 0.02 to 9.7% (Supplementary table S2), we used multiple imputation to generate *m *=* *5 completed datasets.^20^ The associations were estimated in each of the imputed datasets and then these associations from each imputed dataset were combined to account for both the between- and within imputation variations.^20^ All analyses were conducted using SAS version 9.4 (Cary, NC). Statistical tests were two-sided with *P*-values <0.05 indicating statistical significance unless otherwise specified.

## Results

Initial and 6-year changes in characteristics of participants according to quintiles of the LE8 score change are shown in table 1. Participants with the large increase in the LE8 score were more likely to be men, have a lower initial LE8 score, and a lower level of educational attainment (table 1). Supplementary table S3 presents initial characteristics of individual components of the LE8 across quintiles of 6-year changes in the LE8 score.

**Table 1:** Initial (2006) and 6-year changes in characteristics of participants according to quintiles of changes in the LE8 score during the 6-year period (2006–12)

Characteristics	Q1	Q2	Q3	Q4	Q5	*P* for trend*
	Large decrease	Moderate decrease	Relatively stable	Moderate increase	Large increase	
	(*n* = 10 627)	(*n* = 10 721)	(*n* = 10 783)	(*n* = 10 477)	(*n* = 10 755)	
LE8 score						
Initial	73.2 (8.8)	70.4 (9.3)	68.0 (9.8)	64.2 (9.8)	56.5 (10.0)	<0.01
Change	−16.4 (5.2)	−6.9 (1.8)	−1.3 (1.5)	4.5 (1.9)	14.7 (5.7)	<0.01
Age, years						
Initial	49.6 (11.5)	48.5 (11.5)	47.8 (11.7)	48.0 (11.3)	48.8 (10.8)	<0.01
Sex, *n* (%)						
Male	7892 (74.3)	7876 (73.5)	8161 (75.7)	8480 (80.9)	9699 (90.2)	<0.01
BMI, kg/m²						
Initial	24.7 (3.3)	24.8 (3.5)	24.9 (3.5)	25.1 (3.5)	25.6 (3.5)	<0.01
Change	1.0 (2.8)	0.3 (2.4)	−0.0 (2.4)	−0.4 (2.5)	−1.0 (2.8)	<0.01
Alcohol-drinker, *n* (%)						<0.01
Remained a never drinker	5523 (52.0)	5944 (55.4)	5979 (55.4)	5081 (48.5)	3946 (36.7)	
Change from current to former or remained former drinker	1356 (12.8)	1615 (15.1)	1867 (17.3)	2541 (24.3)	4133 (38.4)	
Change from never or past to current drinker or remained current drinker	3748 (35.3)	3162 (29.5)	2937 (27.2)	2855 (27.3)	2676 (24.9)	
Education, *n* (%)						<0.01
Illiteracy or elementary	691 (6.5)	665 (6.2)	677 (6.3)	854 (8.2)	1179 (11.0)	
Middle school	9148 (86.1)	9264 (86.4)	9289 (86.1)	8978 (85.7)	9055 (84.2)	
College or university	788 (7.4)	792 (7.4)	817 (7.6)	645 (6.2)	521 (4.8)	
Scores for each component of LE8						
Diet quality score						
Initial	41.2 (15.6)	39.1 (14.4)	38.7 (14.3)	38.1 (14.7)	36.2 (15.6)	<0.01
Change	−1.1 (19.6)	1.7 (18.7)	2.3 (18.6)	3.7 (19.8)	5.8 (21.0)	<0.01
Physical activity score						
Initial	59.4 (22.2)	55.6 (21.6)	53.7 (22.1)	51.0 (23.9)	44.3 (27.4)	<0.01
Change	−30.9 (34.9)	−17.4 (32.5)	−9.5 (31.4)	−2.5 (33.0)	10.1 (36.4)	<0.01
Smoking score						
Initial	81.3 (37.3)	75.6 (41.2)	69.7 (43.8)	58.5 (47.0)	37.4 (45.6)	<0.01
Change	−13.0 (38.4)	0.3 (34.9)	9.4 (34.5)	20.4 (39.4)	44.9 (44.9)	<0.01
Sleep health score						
Initial	93.2 (15.7)	91.8 (17.4)	90.1 (19.5)	87.0 (21.9)	79.7 (26.5)	<0.01
Change	−8.5 (25.8)	−1.1 (22.3)	2.7 (22.1)	6.6 (23.8)	15.6 (27.4)	<0.01
BMI score						
Initial	71.0 (24.2)	69.7 (24.4)	68.4 (24.6)	67.0 (24.5)	63.4 (24.2)	<0.01
Change	−8.3 (20.9)	−3.0 (19.0)	0.5 (18.9)	3.7 (19.4)	8.8 (20.9)	<0.01
Blood lipids score						
Initial	80.0 (26.0)	77.9 (27.1)	75.4 (27.7)	71.8 (28.6)	65.7 (29.6)	<0.01
Change	−27.0(29.0)	−15.0 (27.2)	−6.7 (26.8)	0.7 (27.9)	11.3 (30.4)	<0.01
Blood glucose score						
Initial	90.8 (20.0)	89.6 (20.9)	87.9 (22.0)	84.8 (23.9)	79.4 (25.8)	<0.01
Change	−17.4(25.0)	−9.7 (23.0)	−4.8 (22.2)	0.1 (23.4)	6.8 (26.1)	<0.01
Blood pressure score						
Initial	68.7 (29.3)	63.6 (32.0)	60.4 (33.2)	55.1 (33.9)	45.9 (34.7)	<0.01
Change	−24.9(31.8)	−11.0 (29.6)	−4.0 (29.7)	3.0 (30.6)	14.1 (33.4)	<0.01

Note: Values are means (SD) for continuous variables or counts (percentages) for categorical variables.

* The *P* for trend was conducted by assigning the median value of each quintile of changes in LE8 score as a continuous variable. A general linear model was used for continuous variables, and a logistic model was used for categorical variables.

### Main results

During a median follow-up of 7.7 years, we documented 4281 incident CVD cases (2167 heart disease and 2320 stroke) (some individuals were diagnosed with both heart disease and stroke). After multivariable adjustments, compared with participants whose LE8 score were relatively stable in a 6-year period, those with the large increase (quintile 5) in a 6-year period had a substantially lower risk of CVD, heart disease and stroke (table 2). The HRs and 95% CIs were 0.67 (0.64, 0.70) for CVD risk, 0.65 (0.61, 0.69) for heart disease risk and 0.71 (0.67, 0.76) for stroke risk (All *P*_trend_ <0.001). In contrast, compared with the participants with relatively stable LE8 score in a 6-year period, those in large decrease of the LE8 score had a 47% (41%, 53%) higher risk of CVD, a 51% (42%, 60%) higher risk of heart disease and a 41% (32%, 49%) higher risk of stroke (All *P*_trend_ < 0.001). When analyzing the continuous data of LE8 score, each 10-point increase of the LE8 score in a 6-year period was associated with a 21–24% lower risk for CVD, heart disease and stroke (table 2).

**Table 2:** Associations of changes in the LE8 score during the 6-year period (2006–12) with subsequent risk of CVD

	Q1	Q2	Q3	Q4	Q5	*P* for trend	Per 10 points increase
	Large decrease	Moderate decrease	Relatively stable	Large increase	Moderate increase		
Mean change in LE8 score	−16.4 (5.2)	−6.9 (1.8)	−1.3 (1.5)	4.5 (1.9)	14.7 (5.7)		
CVD							
No. of case/person-years	924/76 948	790/78 547	794/79 054	812/76 663	961/77 950		
Model 1	1.45 (1.39, 1.52)	1.10 (1.06, 1.15)	1 (Ref)	0.87 (0.83, 0.91)	0.69 (0.66, 0.72)	<0.001	0.78 (0.77, 0.79)
Model 2	1.47 (1.41, 1.53)	1.11 (1.06, 1.16)	1 (Ref)	0.87 (0.83, 0.90)	0.67 (0.64, 0.70)	<0.001	0.77 (0.76, 0.77)
Heart disease							
No. of case/person-years	480/78 393	387/79 880	399/80 285	434/77 924	467/79 525		
Model 1	1.48 (1.40, 1.57)	1.07 (1.01, 1.14)	1 (Ref)	0.92 (0.87, 0.98)	0.67 (0.63, 0.71)	<0.001	0.77 (0.76, 0.79)
Model 2	1.51 (1.42, 1.60)	1.08 (1.02, 1.15)	1 (Ref)	0.92 (0.87, 0.97)	0.65 (0.61, 0.69)	<0.001	0.76 (0.74, 0.77)
Stroke							
No. of case/person-years	489/78 548	432/79 778	433/80 296	415/78 015	551/79 331		
Model 1	1.40 (1.32, 1.48)	1.11 (1.04, 1.18)	1 (Ref)	0.82 (0.77, 0.87)	0.73 (0.69, 0.77)	<0.001	0.80 (0.78, 0.81)
Model 2	1.41 (1.32, 1.49)	1.11 (1.04, 1.17)	1 (Ref)	0.81 (0.77, 0.86)	0.71 (0.67, 0.76)	<0.001	0.79 (0.78, 0.81)

Notes: The data were HRs and 95% CIs; Model 1: Initial age (years in 2006), sex (male, female) and initial LE8 score; Model 2: Model 1 + education (illiteracy or elementary, middle school, college or university), initial income (< median, ≥ median) and changes in income (<median always, ≥median always, change from <median to ≥median, change from ≥median to <median), initial marital status (yes, no) and changes in marital status (always single, always married, change from married to single, change from single to married), initial alcohol-drinker (yes, no) and changes in alcohol-drinker (always drinker, always non-drinker, change from drinker to non-drinker, from non-drinker to drinker), and family history of CVD (yes, no).

**Figure 1:**
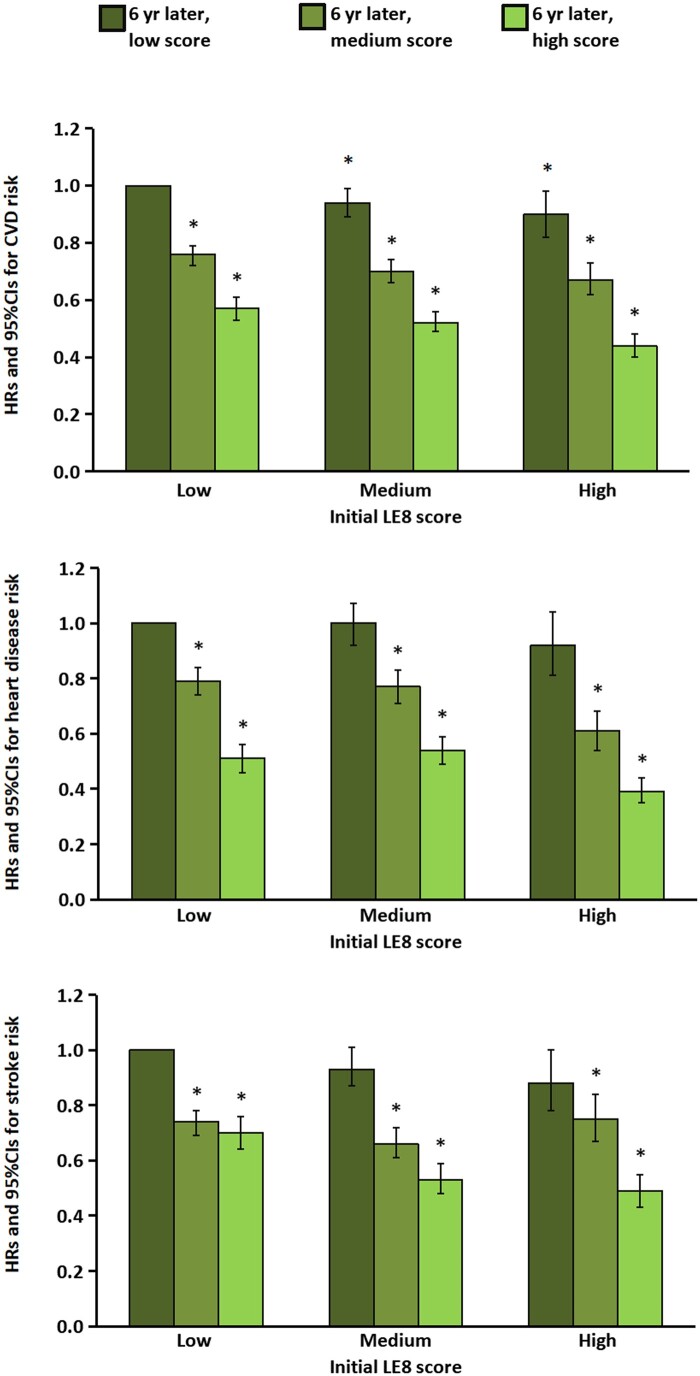
Risk of CVD according to initial LE8 scores in 2006 and scores 6 years later in 2012. Initial LE8 scores are shown as low, medium and high. At 6 years, a participant may have had a consistently low LE8 score over time (reference), a change from a low score to a medium or high score; a consistently medium score over time, a change from a medium score to a low or high score; a consistently high score over time, or a change from a high score to a low or medium score. Multivariable analyses were adjusted for initial age (years in 2006), sex (male, female), initial LE8 score, education (illiteracy or elementary, middle school, college or university), initial income (<median, ≥median) and changes in income (<median always, ≥median always, change from <median to ≥median, change from ≥median to <median), initial marital status (yes, no) and changes in marital status (always single, always married, change from married to single, change from single to married), initial alcohol-drinker (yes, no) and changes in alcohol-drinker (always drinker, always non-drinker, change from drinker to non-drinker, from non-drinker to drinker) and family history of CVD (yes, no).

Restricted cubic spline analyses showed no evidence of nonlinearity for the inverse associations between 6-year changes in the LE8 score with risk of CVD, heart disease, and stroke (all *P*_nonlinearity_ > 0.05) (Supplementary figure S2). When we examined the joint association of scores at the initial cycle and 6 years later, compared with participants who had consistently low LE8 scores over time, participants with the large increase in the LE8 score (low to high) had a 43% lower risk for CVD, a 49% lower risk for heart disease and a 30% lower risk for stroke; whereas those with consistently high LE8 scores (high to high) over time had 51–61% lower risk for CVD, heart disease and stroke (figure 1 and Supplementary table S4).

**Figure 2:**
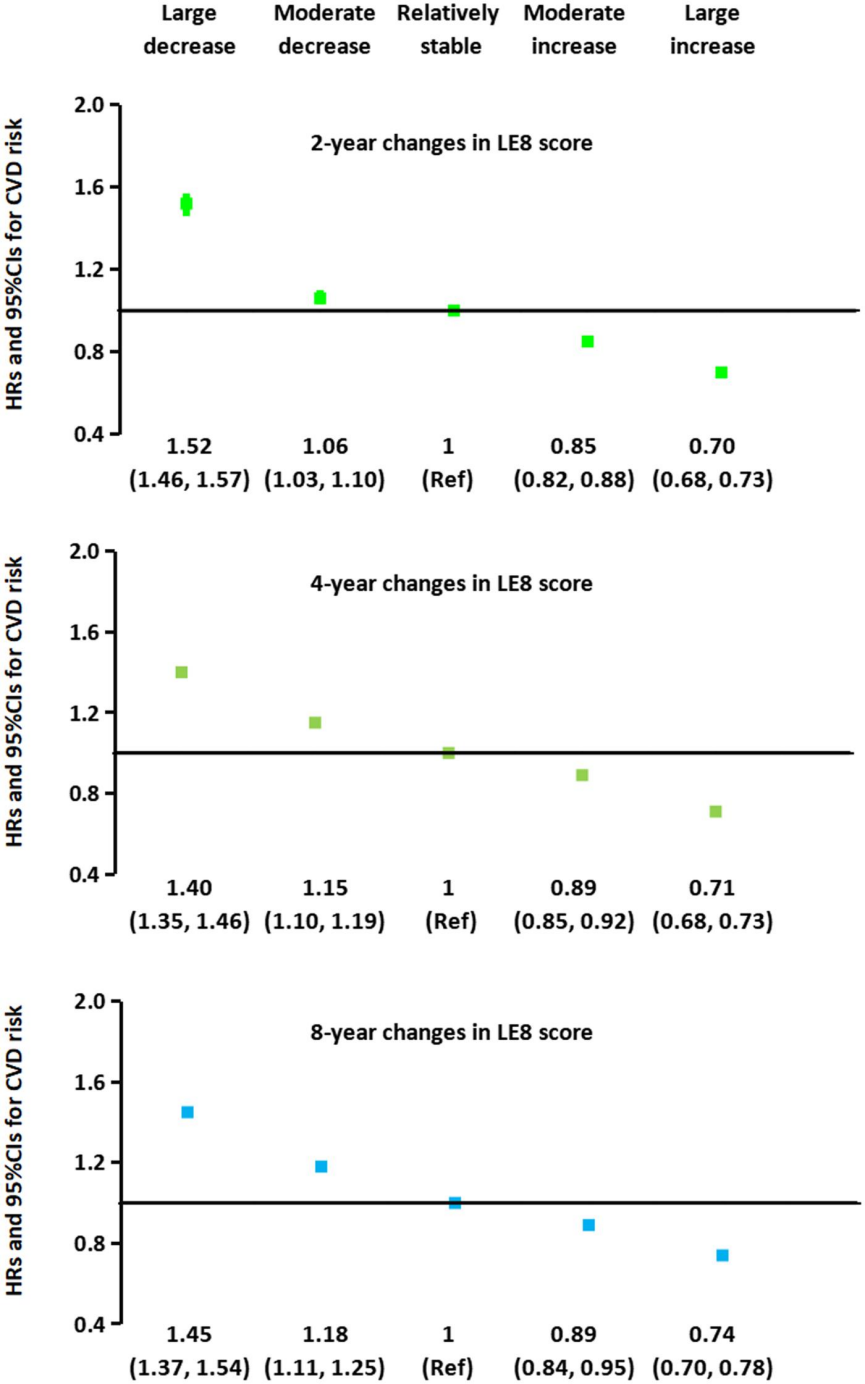
Associations of changes in the LE8 score during the 2-year period (2006–08), the 4-year period (2006–10) and the 8-year period (2006–14) with subsequent risk of CVD. The data were HRs and 95% CIs. Multivariable analyses were adjusted for initial age (years in 2006), sex (male, female), initial LE8 score, education (illiteracy or elementary, middle school, college or university), initial income (<median, ≥median) and changes in income (<median always, ≥median always, change from <median to ≥median, change from ≥median to <median), initial marital status (yes, no) and changes in marital status (always single, always married, change from married to single, change from single to married), initial alcohol- drinker (yes, no) and changes in alcohol-drinker (always drinker, always non-drinker, change from drinker to non-drinker, from non-drinker to drinker) and family history of CVD (yes, no).

In secondary analyses, similar results were observed for 2-year, 4-year and 8-year changes in LE8 score. For example, compared with the participants with stable LE8 scores in the 2, 4, and 8 years, those in the large increase of the LE8 score had a 26–30% lower risk of CVD, while those in the large decrease of the LE8 score had a 40–52% higher risk of CVD (figure 2 and Supplementary table S5).

These inverse associations persisted in subgroup analysis stratified by age, sex, follow-up time, alcohol consumption, initial LE8 score and family history of CVD. None of the interaction tests were statistically significant (All *P*_interaction_ > 0.008, [adjusted for multiple comparisons]) (Supplementary table S6).

### Sensitivity analysis results

In the sensitivity analyses, the significant associations between changes in the LE8 score and risk of CVD, heart disease and stroke remained similar when we added a 2-year lag period after changes in the LE8 score (Supplementary table S7); when we further adjusted for medication use (Supplementary table S8); when we excluded participants with CVD events diagnosed in the first 2 years of the follow-up (Supplementary table S9); or when we excluded participants with cancers at baseline or follow-up (Supplementary table S10). Furthermore, there was a significant trend toward stronger effect sizes of changes in the LE8 score with CVD risk, as compared with those of changes in LS7 score (Supplementary table S11). For example, compared with participants with stable scores, the participants with the large increase and large decrease of the LE8 score had a 33% lower and 47% higher CVD risk, respectively; while those with the large increase and large decrease of the LS7 score had a 31% lower and 40% higher CVD risk, respectively. In addition, increment in each individual score was associated with lower risk of CVD, heart disease or stroke (Supplementary table S12). Finally, the model of changes in LE8 score with adjusted covariates was found to be significant in predicting CVD, heart disease and stroke (Supplementary figure S3). For example, the area under the curve of the 6-year change of LE8 score with the covariates for predicting CVD was 0.72, 95% CI (0.71, 0.73), with the best cut-off being 0.07, and it had a sensitivity of 78% and specificity of 55%.

## Discussion

### Main findings

In the present analyses, we found that compared with participants whose LE8 score remained relatively stable in a 6-year span, those with the large increases of LE8 score had 29–35% lower risk of CVD, heart disease and stroke, while those with the large decrease had 41–51% higher risk of these diseases. In addition, the risk of CVD, heart disease, and stroke was significantly lower (by 51–61%) among participants who maintained a high LE8 score than among those who consistently had a low LE8 score over 6 years. Similar results were observed in shorter-time (2-year, 4-year) and longer-time (8-year) changes in LE8 score.

### Comparison with previous studies

Only a few recent prospective cohort studies have documented the inverse associations of adherence to the LE8 score with risk of CVD events.^4–8^ Also, the previous analyses from our current cohort have indicated that a higher LE8 score is associated with a lower risk of atherosclerotic CVD events among generally healthy people,^21^ a greater number of life years for individuals without CVD,^22^ and a lower risk of mortality among individuals with CVD.^23^ However, most of these previous studies only used baseline LE8 score, which did not capture changes in the LE8 score over time. Understanding how changes in LE8 score are associated with subsequent diabetes risk may allow us to mimic an intervention study where individuals make real-life changes to their adherence to the LE8 score. Leveraging the large sample size, and repeated measurements of the LE8 score and covariates every 2 years, we addressed the main limitations and made some novel observations. We observed that improved LE8 score over 6 years was associated with a substantially lower CVD risk in the following 8 years. Similar results were observed for shorter-time (2-year or 4-year) and longer-time (8-year) changes in the LE8 score. Our results were supported by a previous analysis from our cohort that revealed high baseline LE8 scores and LE8 score trajectories related to lower carotid intima-media thickness, which is an important risk factor of CVD events. Yet, to our knowledge, this is the first study to examine the associations of changes in the LE8 score over time with risk of CVD hard endpoint, including heart disease and stroke.^24^

An important issue in examining changes in the LE8 score and subsequent risk of CVD is to adequately control for initial and concomitant changes in other lifestyle factors (e.g. initial and changes in alcohol intake), socioeconomic factors (e.g. initial and changes in income and marital status). For example, in our study, participants with the large increase of the LE8 score had lower initial LE8 scores. Therefore, we adjusted not only for initial and changes in covariates but also for the initial LE8 score. Furthermore, to consider the ceiling effect of the initial LE8 score, we stratified our analyses by initial LE8 score and observed similar results. These results together suggest that improving LE8 score may lower CVD risk, irrespective of the initial LE8 score. Indeed, our joint analysis of initial and subsequent 6-year LE8 scores also demonstrated that participants with low adherence to the LE8 score in the beginning may still reduce risk of CVD by improving adherence to the LE8 score. Yet, we noticed that compared with those with low LE8 score over time, the participants who maintained a higher LE8 score over time had an even lower risk, highlighting the potential of longer-term adherence to the LE8’s health behaviors as an effective strategy in CVD prevention.

Furthermore, as the LE8 is considered as an advancement to the LS7 to measure and monitor CVH,^2^ we compared the effect sizes of the associations of changes in the LE8 score and the LS7 score with the outcomes. In line with the only previous analysis demonstrating inverse associations of changes in LS7 over time and CVD risk,^25^ our current analyses observed that there was a significant trend for weaker effect sizes of the LS7 score than those of the LE8 score. This could be mainly explained by sleep health, an additional new component of LE8. Indeed, in our sensitivity analysis, we observed that increment in each individual component score of LE8 (e.g. sleep health score, diet quality score and physical activity score) was associated with lower risk of CVD.

### Strengths and limitations

The strengths of this study included the prospective design, the large sample size, the high rate of follow-up, and repeated assessments of diet and lifestyle. However, several limitations should be noted. First, inevitable measurement errors in self-reported dietary and lifestyle assessments led to inaccurate assessment (e.g. non-differential measurement errors), or misclassification bias, which would have tended to underestimate the true associations with the LE8 score.^26^ Second, due to limited data on more specific dietary factors in our cohort, we could not calculate an overall diet quality score, such as alternative healthy eating index to assess diet quality.^27^^,^^28^ Yet, we mainly focused on the LE8 score rather than a diet quality score. We thus used dietary salt, fatty foods and tea consumption to reflect diet quality, which are the most primary dietary factors in relation to CVH among Chinese people.^29^^,^^30^ Third, our study participants were staff of Kailuan Group of Chinese ancestry, which could limit the generalizability of our findings to other racial/ethnic or socioeconomic groups. Finally, it was difficult to rule out residual confounding in observational studies, due to the observational nature of the study design.

In conclusion, among Chinese adults, improving LE8 score in short- and moderate-term was associated with a substantially lower risk of CVD, heart disease and stroke, whereas decreased LE8 score over time was associated with a higher risk of CVD, heart disease and stroke. These findings underscore the importance of improving adherence to the LE8 score to prevent CVD.

## Supplementary Material

ckae063_Supplementary_Data

## Data Availability

Data described in the manuscript, code book and analytic code can be made available upon request pending application and approval from the Kailuan Study (contact E-mail: drwusl@163.com).
