# Supplementary material for: Changes in Life’s Essential 8 and risk of cardiovascular disease in Chinese people
Source: Eur J Public Health. 2024 Apr 4;34(4):766–73. doi: 10.1093/eurpub/ckae063 (PMC11293831; doi:10.1093/eurpub/ckae063)
Supplement: ckae063_Supplementary_Data [file ckae063_supplementary_data.docx]

**Online Supplementary Tables and Figures**

**Changes in Life’s Essential 8 and Risk of Cardiovascular Disease in Chinese People**

| Page 3-6: | **Supplementary Table 1.** Definition of LE8 by AHA and the criteria used in this study |
| --- | --- |
| Page 7: | **Supplementary Table 2.** Missing values of the covariates |
| Page 8-9: | **Supplementary Table 3.** Initial (2006) characteristics of individual components of the LE8 across quintiles of changes in LE8 score during the 6-year period (2006-2012) |
| Page 10: | **Supplementary Table 4.** Hazard ratios (95%CIs) for subsequent risk of cardiovascular disease based on the initial LE8 scores and changes in the LE8 score in 6 years later (From low to low (ref), low to medium, low to high, medium to low, medium to medium, medium to high, high to low, high to medium, high to high) |
| Page 11: | **Supplementary Table 5.** 2-year (2006-2008), 4-year (2006-2010), and 8-year (2006-2014) changes in the LE8 score and subsequent risk of cardiovascular disease |
| Page 12-13: | **Supplementary Table 6.** Stratified hazard ratios (95% CIs) for subsequent risk of cardiovascular disease according to changes in the LE8 score during the 6-year period (2006-2012) by various characteristics of participants |
| Page 14: | **Supplementary Table 7.** A 2-year lagged analysis for associations of changes in the LE8 score during the 6-year period (2006-2012) with subsequent risk of cardiovascular disease |
| Page 15: | **Supplementary Table 8.** Associations of changes in the LE8 score during the 6-year period (2006-2012) with subsequent risk of cardiovascular disease after additional adjustment for medication use |
| Page 16: | **Supplementary Table 9.** Associations of a 6-year changes (2006-2012) in the LE8 score with subsequent risk of cardiovascular disease after excluding cardiovascular disease diagnosed in the first 2 years of follow-up |
| Page 17: | **Supplementary Table 10.** Associations of changes in the LE8 score during the 6-year period (2006-2012) with subsequent risk of cardiovascular disease after excluding cancers at baseline and follow-up |
| Page 18: | **Supplementary Table 11.** Associations of changes in the LS7 score during the 6-year period (2006-2012) with subsequent risk of cardiovascular disease |
| Page 19: | **Supplementary Table 12.** Hazard ratios and 95% CIs of changes in each component score of the LE8 score during the 6-year period (2006-2012) with subsequent risk of cardiovascular disease |
| Page 20: | **Supplementary Figure 1.** Selection of participants in the current analysis |
| Page 21: | **Supplementary Figure 2.** Restricted cubic spline curves for changes in the LE8 score in the 6-year period (2006-2012) and subsequent risk of cardiovascular diseases |
| Page 22: | **Supplementary Figure 3.** Corresponding receiver operating characteristics curves of the model of changes in LE8 score with adjusted covariates for evaluating risk of cardiovascular diseases. |

**Supplementary Table 1. Definition of the LE8 score by AHA and the criteria used in this study.**

| Components | Metric | Method of measurement | Quantification of CVH metric |
| --- | --- | --- | --- |
| Health behaviors | Diet health | Measurement: Self-reported intake of salt, fatty foods, and tea  Examples of salt intake measurement: “What flavor do you prefer?”  Examples of fatty food intake measurement: “How often do you eat fatty foods?”  Example of tea intake measurement: “How often do you drink tea?” | Metric: The unweighted average of salt, fatty food, and tea scoring.  Salt scoring*:  Points Level  100 < 6 g/day  50 6-10 g/day  0 > 10 g/day  Fatty food scoring:  Points Level  100 < 1 time/week  50 1-3 times/week  0 > 3 times/week  Tea scoring:  Points Level  100 ≥ 4 times/week  75 1-3 times/week  50 1-3 times/month  25 < 1 time/month  0 Never |
|  | Physical activity | Measurement: Self-reported times of physical activity per week.  Example tools for measurement: “How many times did you usually spend on physical activity (note: It took at least 20 minutes each time)? ” | Metric: Minutes of physical activity per week.  Scoring:  Points Level  100 ≥ 60  50 20-60  0 < 20 |
|  | Nicotine exposure | Measurement: Self-reported use of cigarettes  Example tools for measurement: Do you smoke cigarettes now? (Never smoker, former smoker, some days, every day) | Metric: Smoking status  Scoring:  Points Status  100 Never smoker  50 Former smokers quit ≥ 1 y  25 Current smokers < 1 cigarette/d  0 Current smoker ≥ 1 cigarette/d |
|  | Sleep health | Measurement: Self-reported average hours of sleep per night  Example tools for measurement: “On average, how many hours of sleep do you get per night?” | Metric: Average hours of sleep per night  Scoring:  Points Level  100 7 - < 9 h  90 9 - < 10 h  70 6 - < 7 h  40 5 - < 6 or ≥ 10 h  20 4 - < 5 h  0 < 4h |
| Health factors | Body mass index | Measurement: Body weight (kg) divided by height squared (m²)  Example tools for measurement: Objective measurement of height and weight | Metric: Body mass index (kg/m^2^)  Scoring:  Points Level  100 < 23  75 23.0-24.9  50 25.0-29.9  25 30.0-34.9  0 ≥ 35.0 |
|  | Blood lipids | Measurement: Plasma total and HDL cholesterol with the calculation of non-HDL cholesterol.  Example tools for measurement: Fasting blood sample.  non-HDL-cholesterol unit conversion:  1mmol/L = 38.67mg/L  1mg/L = 0.02586mmol/L | Metric: Non-HDL cholesterol (mmol/L)  Scoring:  Points Level  100 < 3.36  60 3.36-4.13  40 4.14-4.90  20 4.91-5.68  0 ≥ 5.69  If drug treated, subtract 20 points |
|  | Blood glucose | Measurement: Fasting blood glucose (FBG)  Example tools for measurement: Fasting blood glucose sample.  HBA1C to FBG (mg/L) to conversion:  28.7 * A1C - 46.7 = FBG  FBG unit conversion:  1mg = 0.056mmol/L  1mmol/L = 18.02 mg/dL | Metric: FBG (mmol/L)  Scoring:  Points Level  100 No history of diabetes with FBG < 5.6  60 No diabetes with FBG 5.6-6.9  40 Diabetes with FBG < 8.6  30 Diabetes with FBG 8.6-10.1  20 Diabetes with FBG 10.2-11.6  10 Diabetes with FBG 11.7-13.2  0 Diabetes with FBG ≥ 13.3 |
|  | Blood pressure | Measurement: Appropriately measured systolic and diastolic blood pressure  Example tools for measurement: Corrected Mercury sphygmomanometer | Metric: Systolic and diastolic blood pressure (mm Hg)  Scoring:  Points Level  100 < 120 / < 80  75 120-129 / < 80  50 130-139 or 80-89  25 140-159 or 90-99  0 ≥ 160 or ≥ 100  If drug treated, subtract 20 points. |

LE8, Life’s Essential 8; FBG, fasting blood glucose; HDL cholesterol, high density lipoprotein cholesterol. ^*^According to the Chinese Dietary Guidelines <http://dg.cnsoc.org/zdwynewslist_2101_1.htm> , the light, moderate, and salty flavor refers to <6g/day, 6-12g/day, and >12g/day, respectively

**Supplementary Table 2. Missing values of covariates***

|  | **Missing data** | | | | |
| --- | --- | --- | --- | --- | --- |
|  | **2006** | **2008** | **2010** | **2012** | **2014** |
| **Income** | 0.02% | 9.71% | 0.38% | 8.87% | 8.46% |
| **Alcohol intake** | No missing | 0.02% | 0.01% | 0.02% | 1.74% |
| **Marital status** | No missing | 0.02% | 0.0% | 0.39% | 1.13% |

Values were percentages of missing data for total data of a variable.

**Supplementary Table 3. Initial (2006) characteristics of individual components of the LE8 across quintiles of** **changes in LE8 score during the 6-year period (2006-2012)**

| **Initial characteristics (2006)** | **Q1**  **Large decrease** | **Q2**  **Moderate decrease** | **Q3**  **Relatively stable** | **Q4**  **Moderate increase** | **Q5**  **Large increase** | **P for trend^*^** |
| --- | --- | --- | --- | --- | --- | --- |
|  | (n=10,627) | (n=10,721) | (n=10,783) | (n=10,477) | (n=10,755) |  |
| **Individual components of LE8 score** | |  |  |  |  |  |
| **Tea consumption, n (%)** |  |  |  |  |  | <0.01 |
| Never or less than once/month | 8241 (77.5) | 8634 (80.5) | 8732 (81.0) | 8348 (79.7) | 8574 (79.7) |  |
| 1 to 3 times/month | 680 (6.4) | 657 (6.1) | 663 (6.1) | 687 (6.6) | 737 (6.9) |  |
| 1 to 3 times/week | 617 (5.8) | 509 (4.7) | 535 (5.0) | 519 (5.0) | 524 (4.9) |  |
| ≥4 times/week | 1089 (10.2) | 921 (8.6) | 853 (7.9) | 923 (8.8) | 920 (8.6) |  |
| **Fatty food consumption^*^, n (%)** |  |  |  |  |  | <0.01 |
| <1 times/week | 1129 (10.6) | 890 (8.3) | 845 (7.8) | 824 (7.9) | 824 (7.7) |  |
| 1-3 times/week | 8889 (83.6) | 9066 (84.6) | 9126 (84.6) | 8528 (81.4) | 8253 (76.7) |  |
| >3 times/week | 609 (5.7) | 765 (7.1) | 812 (7.5) | 1125 (10.7) | 1678 (15.6) |  |
| **Salt intake, %** |  |  |  |  |  | <0.01 |
| <6 g/day | 1129 (10.6) | 932 (8.7) | 967 (9.0) | 930 (8.9) | 927 (8.6) |  |
| 6-10 g/day | 8830 (83.1) | 8941 (83.4) | 8835 (81.9) | 8326 (79.5) | 7946 (73.9) |  |
| >10 g/day | 668 (6.3) | 848 (7.9) | 981 (9.1) | 1221 (11.7) | 1882 (17.5) |  |
| **Physical activity level^†^, n (%)** |  |  |  |  |  | <0.01 |
| Never | 237 (2.2) | 466 (4.3) | 686 (6.4) | 1100 (10.5) | 2300 (21.4) |  |
| <3 times/ week | 8149 (76.7) | 8596 (80.2) | 8618 (79.9) | 8072 (77.0) | 7383 (68.6) |  |
| >3 times/week | 2241 (21.1) | 1659 (15.5) | 1479 (13.7) | 1305 (12.5) | 1072 (10.0) |  |
| **Smoke status, n (%)** |  |  |  |  |  | <0.01 |
| Never | 8393 (79.0) | 7820 (72.9) | 7150 (66.3) | 5731 (54.7) | 3540 (32.9) |  |
| Past | 382 (3.6) | 419 (3.9) | 538 (5.0) | 571 (5.5) | 653 (6.1) |  |
| Current | 1852 (17.4) | 2482 (23.2) | 3095 (28.7) | 4175 (39.8) | 6562 (61.0) |  |
| BMI, Kg/m^2^ | 25.0 (3.5) | 24.7 (3.3) | 24.8 (3.5) | 25.1 (3.5) | 25.6 (3.5) |  |
| **Sleep quality, n (%)** |  |  |  |  |  | <0.01 |
| <6 h/day | 374 (3.5) | 497 (4.6) | 679 (6.3) | 960 (9.2) | 1791 (16.7) |  |
| 6-9 h/day | 10161 (95.6) | 10091 (94.1) | 9958 (92.3) | 9342 (89.2) | 8741 (81.3) |  |
| >9 h/day | 92 (0.9) | 133 (1.2) | 146 (1.4) | 175 (1.7) | 223 (2.1) |  |
| **Blood lipid,** **mmol/L** |  |  |  |  |  |  |
| TC | 4.8 (1.0) | 4.8 (1.1) | 4.9 (1.1) | 4.9 (1.2) | 5.1 (1.4) | <0.01 |
| HDL cholesterol | 1.6 (0.4) | 1.6 (0.4) | 1.5 (0.4) | 1.5 (0.4) | 1.5 (0.4) | <0.01 |
| **Blood glucose, mmol/L** | 5.2 (1.3) | 5.3 (1.3) | 5.3 (1.4) | 5.5 (1.6) | 5.7 (1.8) | <0.01 |
| **Blood pressure, mmHg** |  |  |  |  |  |  |
| SBP | 124.1 (17.5) | 126.2 (19.2) | 127.6 (19.5) | 130.2 (20.0) | 134.9 (20.1) | <0.01 |
| DBP | 80.1 (10.1) | 81.4 (11.1) | 82.3 (11.2) | 84.0 (11.6) | 86.9 (11.8) | <0.01 |

Values are means (standard deviations) for continuous variables or counts (percentages) for categorical variables. LE8, Life’s Essential 8; SBP, systolic blood pressure, DBP; diastolic blood pressure, BMI; body mass index, TC; total cholesterol, HDL cholesterol; high density lipoprotein cholesterol. ^*^Fatty foods included red and processed meat, fried foods, fast foods, fatty snacks, animal-fat foods and so on in the Kailuan Study. ^†^It took at least 20 minutes each time. ^*^The P for trend was conducted by assigning the median value of each quintile of changes in LE8 score as a continuous variable. A general linear model was used for continuous variables, and a logistic model was used for categorical variables.**Supplementary Table 4. Hazard ratios (95%CIs) for subsequent risk of cardiovascular disease based on the initial LE8 scores and changes in the LE8 score in 6 years later (From low to low (ref), low to medium, low to high, medium to low, medium to medium, medium to high, high to low, high to medium, high to high)**

| **LE8 score** | **Low-low** | **Low-Medium** | **Low-High** | **Medium-Low** | **Medium-Medium** | **Medium-High** | **High-low** | **High-Medium** | **High- High** |
| --- | --- | --- | --- | --- | --- | --- | --- | --- | --- |
| **CVD** | 1 (Ref) | 0.76  (0.72, 0.79) | 0.57  (0.53, 0.61) | 0.94  (0.89, 0.99) | 0.70  (0.66, 0.74) | 0.52  (0.49, 0.56) | 0.90  (0.82, 0.98) | 0.67  (0.62, 0.73) | 0.44  (0.40, 0.48) |
| **Heart disease** | 1 (Ref) | 0.79  (0.74, 0.84) | 0.51  (0.46, 0.56) | 1.00  (0.92, 1.07) | 0.77  (0.71, 0.83) | 0.54  (0.49, 0.59) | 0.92  (0.81, 1.04) | 0.61  (0.54, 0.68) | 0.39  (0.35, 0.44) |
| **Stroke** | 1 (Ref) | 0.74  (0.69, 0.78) | 0.70  (0.64, 0.76) | 0.93  (0.87, 1.01) | 0.66  (0.61, 0.72) | 0.53  (0.48, 0.59) | 0.88  (0.78, 1.00) | 0.75  (0.67, 0.84) | 0.49  (0.43, 0.55) |

The data were HRs and 95% CIs. Multivariable analyses were adjusted for initial age (years in 2006), sex (male, female), initial LE8 score, education (illiteracy or elementary, middle school, college or university), initial income (< median, ≥ median) and changes in income (< median always, ≥ median always, change from < median to ≥ median, change from ≥ median to < median), initial marital status (yes, no) and changes in marital status (always single, always married, change from married to single, change from single to married), initial alcohol-drinker (yes, no) and changes in alcohol-drinker (always drinker, always non-drinker, change from drinker to non-drinker, from non-drinker to drinker), and family history of CVD (yes, no). LE8, Life’s Essential 8; CVD, cardiovascular disease.

**Supplementary Table 5. 2-year (2006-2008), 4-year (2006-2010), and 8-year (2006-2014) changes in the LE8 score and subsequent risk of cardiovascular disease**

| **LE8 score** | **Q1**  **Large decrease** | **Q2**  **Moderate decrease** | **Q3**  **Relatively stable** | **Q4**  **Moderate increase** | **Q5**  **Large increase** | **Per 10 points increase** |
| --- | --- | --- | --- | --- | --- | --- |
| **2-year changes** |  |  |  |  |  |  |
| CVD | 1.52 (1.46, 1.57) | 1.06 (1.03, 1.10) | 1 (Ref) | 0.85 (0.82, 0.88) | 0.70 (0.68, 0.73) | 0.78 (0.77, 0.79) |
| Heart disease | 1.53 (1.46, 1.60) | 1.05 (1.00, 1.10) | 1 (Ref) | 0.83 (0.79, 0.88) | 0.69 (0.66, 0.73) | 0.78 (0.77, 0.79) |
| Stroke | 1.45 (1.38, 1.52) | 1.06 (1.01, 1.12) | 1 (Ref) | 0.84 (0.80, 0.89) | 0.72 (0.69, 0.76) | 0.80 (0.79, 0.81) |
| **4-year changes** |  |  |  |  |  |  |
| CVD | 1.40 (1.35, 1.46) | 1.15 (1.10, 1.19) | 1 (Ref) | 0.89 (0.85, 0.92) | 0.71 (0.68, 0.73) | 0.79 (0.78, 0.80) |
| Heart disease | 1.35 (1.29, 1.43) | 1.10 (1.04, 1.16) | 1 (Ref) | 0.91 (0.87, 0.96) | 0.70 (0.66, 0.74) | 0.80 (0.79, 0.82) |
| Stroke | 1.49 (1.41, 1.57) | 1.21 (1.15, 1.28) | 1 (Ref) | 0.89 (0.85, 0.94) | 0.75 (0.71, 0.79) | 0.79 (0.77, 0.80) |
| **8-year changes** |  |  |  |  |  |  |
| CVD | 1.45 (1.37, 1.54) | 1.18 (1.11, 1.25) | 1 (Ref) | 0.89 (0.84, 0.95) | 0.74 (0.70, 0.78) | 0.79 (0.77, 0.80) |
| Heart disease | 1.35 (1.25, 1.46) | 0.98 (0.90, 1.06) | 1 (Ref) | 0.79 (0.73, 0.86) | 0.68 (0.63, 0.74) | 0.80 (0.78, 0.82) |
| Stroke | 1.52 (1.40, 1.64) | 1.32 (1.22, 1.43) | 1 (Ref) | 0.97 (0.89, 1.05) | 0.77 (0.71, 0.84) | 0.78 (0.76, 0.80) |

The data were HRs and 95% CIs. Multivariable analyses were adjusted for initial age (years in 2006), sex (male, female), initial LE8 score, education (illiteracy or elementary, middle school, college or university), initial income (< median, ≥ median) and changes in income (< median always, ≥ median always, change from < median to ≥ median, change from ≥ median to < median), initial marital status (yes, no) and changes in marital status (always single, always married, change from married to single, change from single to married), initial alcohol-drinker (yes, no) and changes in alcohol-drinker (always drinker, always non-drinker, change from drinker to non-drinker, from non-drinker to drinker), and family history of CVD (yes, no). LE8, Life’s Essential 8; CVD, cardiovascular disease.

**Supplementary Table 6. Stratified hazard ratios (95% CIs) for subsequent risk of cardiovascular disease according to changes in the LE8 score during the 6-year period (2006-2012) by various characteristics of participants**

| **Life’ essential 8 score** | | | | | | | |
| --- | --- | --- | --- | --- | --- | --- | --- |
|  | **Q1**  **Large decrease** | **Q2**  **Moderate decrease** | **Q3**  **Relatively stable** | **Q4**  **Moderate increase** | **Q5**  **Large increase** | **Per 10 points increase** | **P_interaction_** |
| **Age** |  |  |  |  |  |  |  |
| <65 years | 1.55 (1.48, 1.63) | 1.10 (1.05, 1.16) | 1 (Ref) | 0.87 (0.83, 0.91) | 0.67 (0.64, 0.70) | 0.75 (0.74, 0.76) | 0.18 |
| ≥65 years | 1.10 (0.99, 1.22) | 1.06 (0.95, 1.18) | 1 (Ref) | 0.89 (0.80, 1.00) | 0.73 (0.64, 0.82) | 0.88 (0.84, 0.91) |  |
| **Sex** |  |  |  |  |  |  |  |
| Female | 1.69 (1.52, 1.88) | 1.09 (0.98, 1.22) | 1 (Ref) | 0.94 (0.84, 1.06) | 0.57 (0.49, 0.66) | 0.70 (0.67, 0.73) | 0.64 |
| Male | 1.44 (1.37, 1.51) | 1.11 (1.06, 1.16) | 1 (Ref) | 0.86 (0.82, 0.90) | 0.69 (0.66, 0.72) | 0.78 (0.77, 0.79) |  |
| **BMI at baseline (2012)** |  |  |  |  |  |  |  |
| <25kg/m^2^ | 1.47 (1.38, 1.58) | 1.12 (1.04, 1.19) | 1 (Ref) | 0.92 (0.86, 0.98) | 0.67 (0.63, 0.72) | 0.77 (0.75, 0.78) | 0.84 |
| ≥25kg/m^2^ | 1.46 (1.38, 1.55) | 1.09 (1.02, 1.15) | 1 (Ref) | 0.82 (0.77, 0.88) | 0.67 (0.63, 0.72) | 0.76 (0.74, 0.78) |  |
| **Alcohol-drinker at baseline (2012)** | |  |  |  |  |  |  |
| No (Never) | 1.46 (1.38, 1.54) | 1.10 (1.05, 1.16) | 1 (Ref) | 0.88 (0.83, 0.92) | 0.64 (0.61, 0.68) | 0.76 (0.75, 0.78) | 0.64 |
| Yes (Past, or current) | 1.50 (1.38, 1.62) | 1.10 (1.01, 1.20) | 1 (Ref) | 0.84 (0.77, 0.91) | 0.75 (0.68, 0.82) | 0.77 (0.75, 0.79) |  |
| **Smoker at baseline (2012)** |  |  |  |  |  |  |  |
| No (Never) | 1.33 (1.26, 1.41) | 1.11 (1.05, 1.17) | 1 (Ref) | 0.90 (0.85, 0.95) | 0.66 (0.62, 0.70) | 0.78 (0.77, 0.80) | 0.03 |
| Yes (Past, or current) | 1.62 (1.51, 1.74) | 1.09 (1.01, 1.18) | 1 (Ref) | 0.82 (0.75, 0.88) | 0.72 (0.66, 0.78) | 0.74 (0.73, 0.76) |  |
| **Family history of CVD** |  |  |  |  |  |  |  |
| No | 1.47 (1.40, 1.53) | 1.10 (1.05, 1.15) | 1 (Ref) | 0.87 (0.83, 0.91) | 0.67 (0.64, 0.70) | 0.77 (0.76, 0.78) | 0.29 |
| Yes | 1.57 (1.04, 2.36) | 1.54 (1.02, 2.33) | 1 (Ref) | 0.40 (0.23, 0.69) | 0.50 (0.30, 0.84) | 0.64 (0.55, 0.74) |  |
| **Initial LE8 score (2006)** |  |  |  |  |  |  |  |
| <Median | 1.47 (1.38, 1.56) | 1.13 (1.07, 1.20) | 1 (Ref) | 0.86 (0.82, 0.91) | 0.69 (0.66, 0.73) | 0.78 (0.76, 0.79) | 0.42 |
| ≥Median | 1.47 (1.38, 1.57) | 1.06 (0.99, 1.14) | 1 (Ref) | 0.90 (0.82, 0.98) | 0.62 (0.54, 0.71) | 0.76 (0.74, 0.78) |  |
| **Follow-up time** |  |  |  |  |  |  |  |
| ≤Median | 1.51 (1.45, 1.58) | 1.11 (1.06, 1.16) | 1 (Ref) | 0.88 (0.84, 0.92) | 0.71 (0.68, 0.74) | 0.77 (0.76, 0.78) | 0.09 |
| >Median | 1.38 (0.95, 2.00) | 1.77 (1.25, 2.51) | 1 (Ref) | 1.33 (0.93, 1.91) | 0.39 (0.24, 0.62) | 0.72 (0.64, 0.81) |  |

The data were HRs and 95% CIs. Multivariable analyses were adjusted for initial age (years in 2006), sex (male, female), initial LE8 score, education (illiteracy or elementary, middle school, college or university), initial income (< median, ≥ median) and changes in income (< median always, ≥ median always, change from < median to ≥ median, change from ≥ median to < median), initial marital status (yes, no) and changes in marital status (always single, always married, change from married to single, change from single to married), initial alcohol-drinker (yes, no) and changes in alcohol-drinker (always drinker, always non-drinker, change from drinker to non-drinker, from non-drinker to drinker), and family history of CVD (yes, no). P_interaction_ was calculated using the Wald test by including interactions between changes in the LE8 score and each of the following variables: age, sex, BMI, smoker, alcohol drink, initial LE8 score, family history of CVD, and follow-up time. Given the potential for multiple testing, the statistical level for significance was set at 0.008 (0.05/6 comparisons). LE8, Life’s Essential 8; CVD, cardiovascular disease.**Supplementary Table 7. A 2-year lagged analysis for associations of changes in the LE8 score during the 6-year period (2006-2012) with subsequent risk of cardiovascular disease**

| **Life’s essential 8 scores** | **Q1**  **Large decrease** | **Q2**  **Moderate decrease** | **Q3**  **Relatively stable** | **Q4**  **Moderate increase** | **Q5**  **Large increase** | **Per 10 points increase** |
| --- | --- | --- | --- | --- | --- | --- |
| **CVD** | 1.39 (1.33, 1.46) | 1.08 (1.03, 1.13) | 1 (Ref) | 0.88 (0.84, 0.92) | 0.68 (0.64, 0.71) | 0.78 (0.77, 0.80) |
| **Heart disease** | 1.48 (1.39, 1.59) | 1.08 (1.01, 1.16) | 1 (Ref) | 0.96 (0.90, 1.03) | 0.64 (0.59, 0.69) | 0.77 (0.75, 0.79) |
| **Stroke** | 1.30 (1.22, 1.39) | 1.06 (0.99, 1.13) | 1 (Ref) | 0.81 (0.76, 0.86) | 0.73 (0.68, 0.78) | 0.81 (0.80, 0.83) |

The data were HRs and 95% CIs. Multivariable analyses were adjusted for initial age (years in 2006), sex (male, female), initial LE8 score, education (illiteracy or elementary, middle school, college or university), initial income (< median, ≥ median) and changes in income (< median always, ≥ median always, change from < median to ≥ median, change from ≥ median to < median), initial marital status (yes, no), and changes in marital status (always single, always married, change from married to single, change from single to married), initial alcohol-drinker (yes, no), changes in alcohol-drinker (always drinker, always non-drinker, change from drinker to non-drinker, from non-drinker to drinker), and family history of CVD (yes, no). LE8, Life’s Essential 8; CVD, cardiovascular disease.

**Supplementary Table 8. Associations of changes in the LE8 score during the 6-year period (2006-2012) with subsequent risk of cardiovascular disease after additional adjustment for medication use**

| **Life’s essential 8 scores** | **Q1**  **Large decrease** | **Q2**  **Moderate decrease** | **Q3**  **Relatively stable** | **Q4**  **Moderate increase** | **Q5**  **Large increase** | **Per 10 points increase** |
| --- | --- | --- | --- | --- | --- | --- |
| **CVD** | 1.46 (1.39, 1.52) | 1.10 (1.06, 1.15) | 1 (Ref) | 0.87 (0.83, 0.90) | 0.68 (0.65, 0.71) | 0.77 (0.76, 0.78) |
| **Heart disease** | 1.50 (1.41, 1.59) | 1.07 (1.01, 1.14) | 1 (Ref) | 0.92 (0.87, 0.97) | 0.66 (0.62, 0.70) | 0.76 (0.75, 0.78) |
| **Stroke** | 1.39 (1.31, 1.48) | 1.11 (1.04, 1.17) | 1 (Ref) | 0.81 (0.77, 0.87) | 0.72 (0.68, 0.77) | 0.80 (0.78, 0.81) |

The data were HRs and 95% CIs. Multivariable analyses were adjusted for initial age (years in 2006), sex (male, female), initial LE8 score, education (illiteracy or elementary, middle school, college or university), initial income (< median, ≥ median) and changes in income (< median always, ≥ median always, change from < median to ≥ median, change from ≥ median to < median), initial marital status (yes, no) and changes in marital status (always single, always married, change from married to single, change from single to married), initial alcohol-drinker (yes, no) and changes in alcohol-drinker (always drinker, always non-drinker, change from drinker to non-drinker, from non-drinker to drinker), family history of CVD (yes, no), antihypertensive medication use, lipid-lowering medication use, and glucose-lowering medication use. LE8, Life’s Essential 8; CVD, cardiovascular disease.

**Supplementary Table 9. Associations of a 6-year changes (2006-2012) in the LE8 score with subsequent risk of cardiovascular disease after excluding cardiovascular disease events diagnosed in the first 2 years of follow-up**

| **Life’s essential 8 scores** | **Q1**  **Large decrease** | **Q2**  **Moderate decrease** | **Q3**  **Relatively stable** | **Q4**  **Moderate increase** | **Q5**  **Large increase** | **Per 10 points increase** |
| --- | --- | --- | --- | --- | --- | --- |
| **CVD** | 1.43 (1.36, 1.49) | 1.09 (1.04, 1.15) | 1 (Ref) | 0.87 (0.83, 0.91) | 0.68 (0.65, 0.71) | 0.78 (0.76, 0.79) |
| **Heart disease** | 1.49 (1.40, 1.59) | 1.06 (1.00, 1.13) | 1 (Ref) | 0.94 (0.88, 1.00) | 0.64 (0.60, 0.69) | 0.76 (0.75, 0.78) |
| **Stroke** | 1.35 (1.27, 1.44) | 1.11 (1.04, 1.19) | 1 (Ref) | 0.81 (0.76, 0.86) | 0.73 (0.69, 0.78) | 0.80 (0.79, 0.82) |

The data were HRs and 95% CIs. Multivariable analyses were adjusted for initial age (years in 2006), sex (male, female), initial LE8 score, education (illiteracy or elementary, middle school, college or university), initial income (< median, ≥ median) and changes in income (< median always, ≥ median always, change from < median to ≥ median, change from ≥ median to < median), initial marital status (yes, no) and changes in marital status (always single, always married, change from married to single, change from single to married), initial alcohol-drinker (yes, no) and changes in alcohol-drinker (always drinker, always non-drinker, change from drinker to non-drinker, from non-drinker to drinker), and family history of CVD (yes, no). LE8, Life’s Essential 8; CVD, cardiovascular disease.

**Supplementary Table 10. Associations of changes in the LE8 score during the 6-year period (2006-2012) with subsequent risk of cardiovascular disease after excluding cancers at baseline and follow-up.**

| **Life’s essential 8 scores** | **Q1**  **Large decrease** | **Q2**  **Moderate decrease** | **Q3**  **Relatively stable** | **Q4**  **Moderate increase** | **Q5**  **Large increase** | **Per 10 points increase** |
| --- | --- | --- | --- | --- | --- | --- |
| **CVD** | 1.46 (1.40, 1.53) | 1.09 (1.05, 1.14) | 1 (Ref) | 0.87 (0.83, 0.91) | 0.66 (0.63, 0.69) | 0.76 (0.75, 0.78) |
| **Heart disease** | 1.50 (1.41, 1.60) | 1.06 (1.00, 1.13) | 1 (Ref) | 0.92 (0.87, 0.98) | 0.65 (0.61, 0.69) | 0.76 (0.74, 0.77) |
| **Stroke** | 1.40 (1.31, 1.48) | 1.09 (1.03, 1.16) | 1 (Ref) | 0.82 (0.77, 0.87) | 0.70 (0.66, 0.74) | 0.79 (0.77, 0.80) |

The data were HRs and 95 %CIs. Multivariable analyses were adjusted for initial age (years in 2006), sex (male, female), initial LE8 score, education (illiteracy or elementary, middle school, college or university), initial income (< median, ≥ median) and changes in income (< median always, ≥ median always, change from < median to ≥ median, change from≥ median to < median), initial marital status (yes, no) and changes in marital status (always single, always married, change from married to single, change from single to married), initial alcohol-drinker (yes, no) and changes in alcohol-drinker (always drinker, always non-drinker, change from drinker to non-drinker, from non-drinker to drinker), and family history of CVD (yes, no). LE8, Life’s Essential 8; CVD, cardiovascular disease.

**Supplementary Table 11. Associations of changes in the LS7 score during the 6-year period (2006-2012) with subsequent risk of cardiovascular disease**

| **LS7 score** | **Q1**  **Large decrease** | **Q2**  **Moderate decrease** | **Q3**  **Relatively stable** | **Q4**  **Moderate increase** | **Q5**  **Large increase** |
| --- | --- | --- | --- | --- | --- |
| **CVD** | 1.40 (1.34, 1.46) | 1.19 (1.14, 1.24) | 1 (Ref) | 0.88 (0.84, 0.92) | 0.69 (0.66, 0.73) |
| **Heart disease** | 1.42 (1.34, 1.50) | 1.10 (1.03, 1.16) | 1 (Ref) | 0.88 (0.83, 0.93) | 0.65 (0.61, 0.70) |
| **Stroke** | 1.37 (1.29, 1.45) | 1.30 (1.23, 1.37) | 1 (Ref) | 0.90 (0.85, 0.96) | 0.77 (0.72, 0.82) |

The data were HRs and 95 %CIs. Multivariable analyses were adjusted for initial age (years in 2006), sex (male, female), initial LE8 score, education (illiteracy or elementary, middle school, college or university), initial income (< median, ≥ median) and changes in income (< median always, ≥ median always, change from < median to ≥ median, change from≥ median to < median), initial marital status (yes, no) and changes in marital status (always single, always married, change from married to single, change from single to married), initial alcohol-drinker (yes, no) and changes in alcohol-drinker (always drinker, always non-drinker, change from drinker to non-drinker, from non-drinker to drinker), family history of CVD (yes, no), and sleep health (quintiles). LS7, Life’s Simple 7; CVD, cardiovascular disease.

**Supplementary Table 12. Hazard ratios and 95% CIs of changes in each component score of the LE8 score during the 6-year period (2006-2012) with subsequent risk of cardiovascular disease**

| **Each 20-point increase^*^** | **CVD** | **Heart disease** | **Stroke** |
| --- | --- | --- | --- |
| **Score for diet quality** | 0.91 (0.90, 0.92) | 0.93 (0.91, 0.94) | 0.90 (0.88, 0.92) |
| **Score for physical activity** | 0.96 (0.95, 0.97) | 0.95 (0.94, 0.96) | 0.97 (0.96, 0.98) |
| **Score for smoking** | 0.91 (0.91, 0.92) | 0.92 (0.91, 0.93) | 0.91 (0.90, 0.92) |
| **Score for sleep health** | 0.92 (0.91, 0.93) | 0.91 (0.90, 0.92) | 0.94 (0.92, 0.95) |
| **Score for BMI** | 0.96 (0.95, 0.97) | 0.94 (0.93, 0.96) | 0.97 (0.96, 0.99) |
| **Score for blood lipids** | 0.92 (0.91, 0.92) | 0.91 (0.90, 0.92) | 0.93 (0.92, 0.94) |
| **Score for blood glucose** | 0.94 (0.94, 0.95) | 0.93 (0.92, 0.95) | 0.96 (0.95, 0.97) |
| **Score for blood pressure** | 0.95 (0.95, 0.96) | 0.96 (0.95, 0.97) | 0.95 (0.94, 0.96) |

The data were HRs and 95% CIs. Multivariable analyses were adjusted for initial age (years in 2006), sex (male, female), initial LE8 score, education (illiteracy or elementary, middle school, college or university), initial income (< median, ≥ median) and changes in income (< median always, ≥ median always, change from < median to ≥ median, change from≥ median to < median), initial marital status (yes, no) and changes in marital status (always single, always married, change from married to single, change from single to married), initial alcohol-drinker (yes, no) and changes in alcohol-drinker (always drinker, always non-drinker, change from drinker to non-drinker, from non-drinker to drinker), and family history of CVD (yes, no). Mutual adjustment was conducted for the 8 individual component scores. ^*^Higher diet quality score, higher physical activity score, higher smoking score, higher sleep health score, higher BMI score, higher blood lipids score, higher blood glucose score, and higher blood pressure score reflected higher diet quality, more physical activity, less smoking, higher sleep health quality, lower BMI, lower blood non-HDL cholesterol, lower blood glucose, and lower blood pressure, respectively. LE8, Life’s Essential 8; CVD, cardiovascular disease.

**
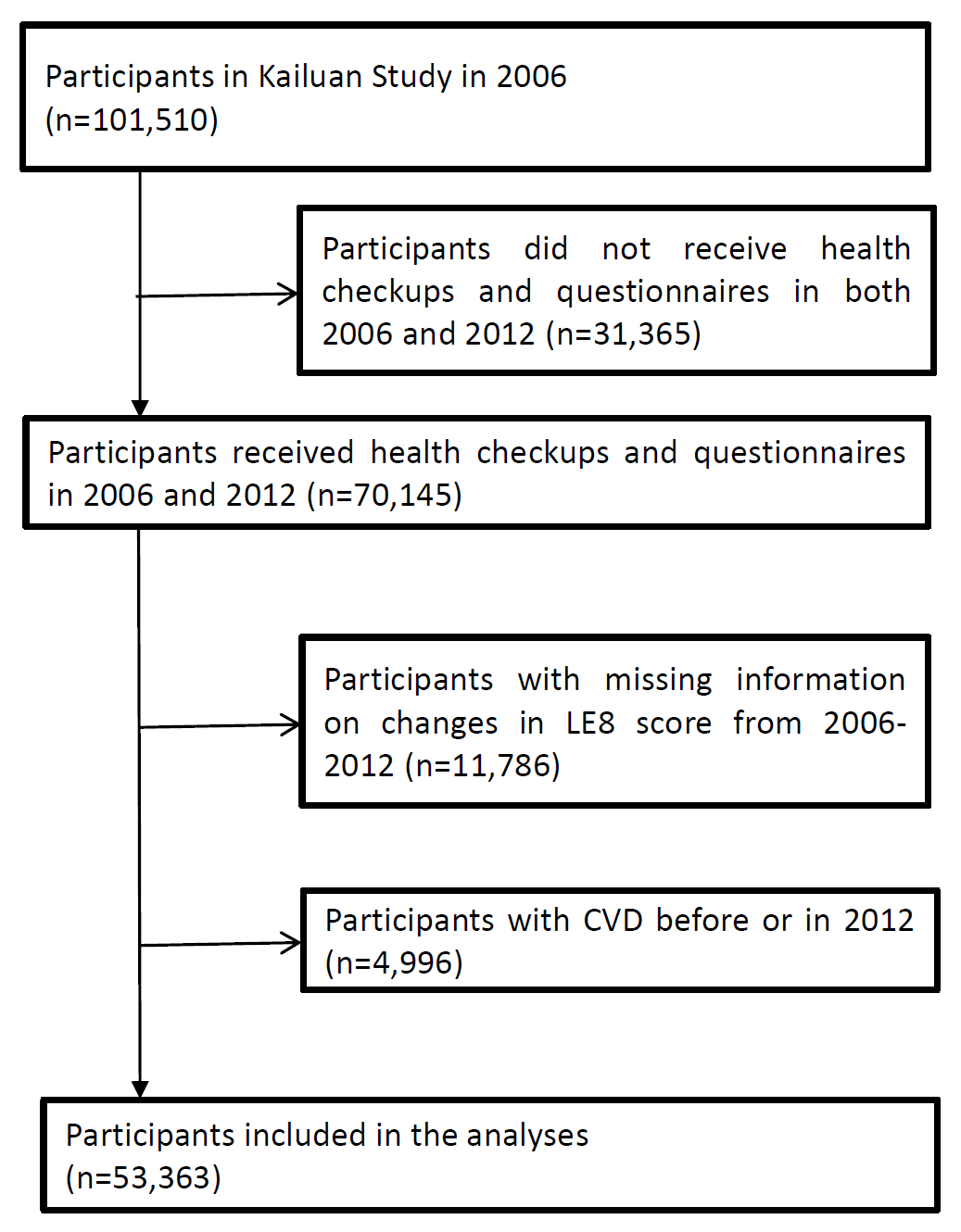
**

**Supplementary Figure 1. Selection of participants in the current analyses**

**
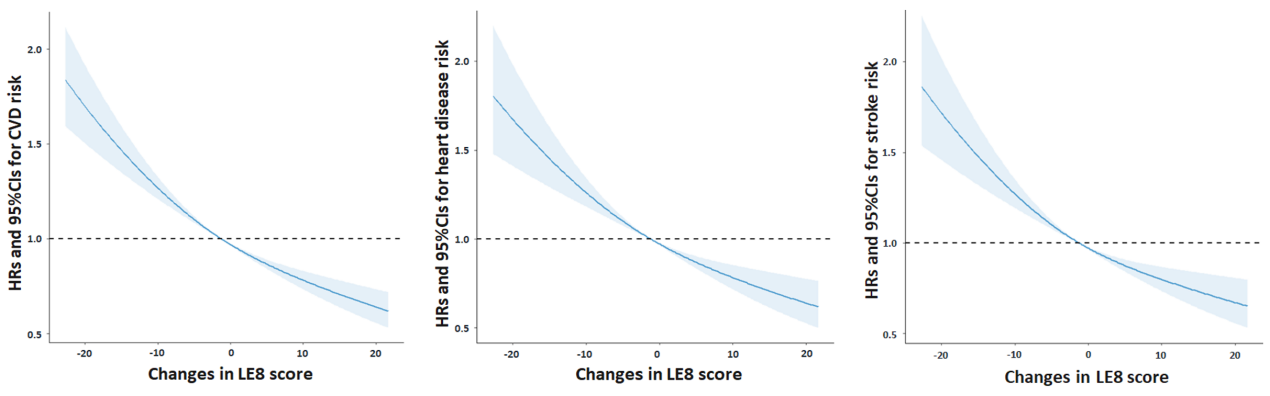
**

**Supplementary Figure 2. Restricted cubic spline curves for changes in the LE8 score in the 6-year period (2006-2012) and subsequent risk of cardiovascular diseases.**

P values for non-linearity for the associations of changes in the LE8 score with subsequent risk of CVD, heart disease, and stroke were 0.29, 0.43, and 0.29, respectively. Multivariable-adjusted hazard ratios are calculated by restricted cubic spline regression adjusted for initial age (years in 2006), sex (male, female), initial LE8 score, education (illiteracy or elementary, middle school, college or university), initial income (< median, ≥ median) and changes in income (< median always, ≥ median always, change from < median to ≥ median, change from ≥ median to < median), initial marital status (yes, no) and changes in marital status (always single, always married, change from married to single, change from single to married), initial alcohol-drinker (yes, no) and changes in alcohol-drinker (always drinker, always non-drinker, change from drinker to non-drinker, from non-drinker to drinker), and family history of CVD (yes, no). LE8, Life’s Essential 8; CVD, cardiovascular disease.


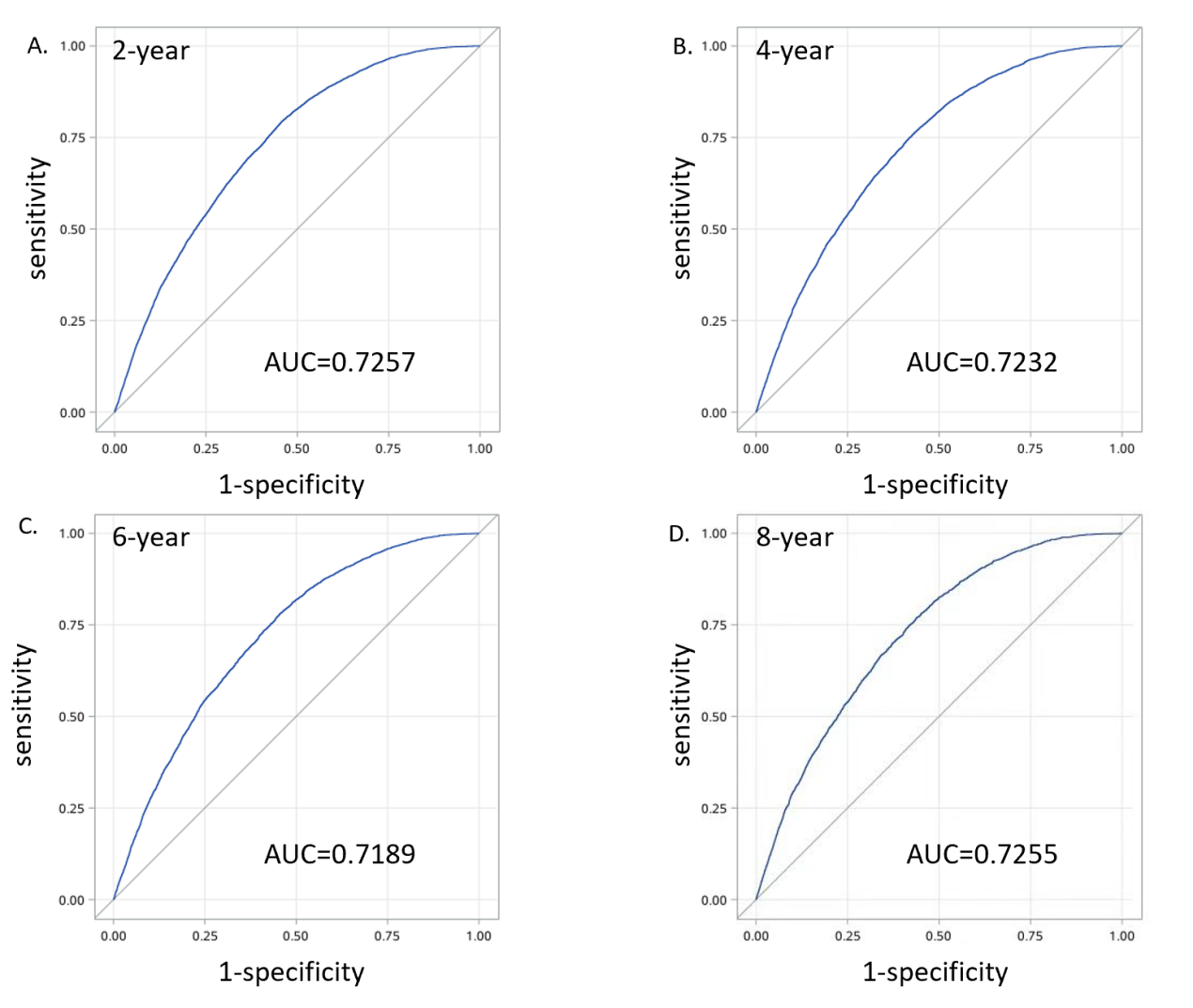


**Supplementary Figure 3. Corresponding receiver operating characteristics curves of the model of changes in LE8 score with adjusted covariates for evaluating risk of cardiovascular diseases.**

The analyses were conducted based the multivariate model, including changes in LE8 score (exposure), initial age (years in 2006), sex (male, female), initial LE8 score, education (illiteracy or elementary, middle school, college or university), initial income (< median, ≥ median) and changes in income (< median always, ≥ median always, change from < median to ≥ median, change from≥ median to < median), initial marital status (yes, no) and changes in marital status (always single, always married, change from married to single, change from single to married), initial alcohol-drinker (yes, no) and changes in alcohol-drinker (always drinker, always non-drinker, change from drinker to non-drinker, from non-drinker to drinker), and family history of CVD (yes, no).

A. The AUC of the 2-year change of LE8 score is 0.73 (0.72, 0.73), with the best cut-off being 0.09, and it had a sensitivity of 79% and specificity of 54%. B. The AUC of the 4-year change is 0.72 (0.72, 0.73), with the best cut-off being 0.08, and it had a sensitivity of 75% and specificity of 54%. C. The AUC of the 6-year change is 0.72 (0.71, 0.73), with the best cut-off being 0.07, and it had a sensitivity of 78% and specificity of 55%. D. The AUC of the 8-year change is 0.73 (0.72, 0.73), with the best cut-off being 0.05, and it had a sensitivity of 77% and specificity of 56%.

AUC, Area under the curve; LE8, Life’s Essential 8; CVD, cardiovascular disease.
